# SUPPORT Tools for evidence-informed health Policymaking (STP) 4: Using research evidence to clarify a problem

**DOI:** 10.1186/1478-4505-7-S1-S4

**Published:** 2009-12-16

**Authors:** John N Lavis, Michael G Wilson, Andrew D Oxman, Simon Lewin, Atle Fretheim

**Affiliations:** 1 Centre for Health Economics and Policy Analysis, Department of Clinical Epidemiology and Biostatistics, and Department of Political Science, McMaster University, 1200 Main St. West, HSC-2D3, Hamilton, ON, Canada, L8N 3Z5; 2 Health Research Methodology PhD Program and Department of Clinical Epidemiology and Biostatistics, 1200 Main St. West, HSC-2D1 Area, Hamilton, ON, Canada, L8N 3Z5; 3 Norwegian Knowledge Centre for the Health Services, P.O. Box 7004, St. Olavs plass, N-0130 Oslo, Norway; 4 Norwegian Knowledge Centre for the Health Services, P.O. Box 7004, St. Olavs plass, N-0130 Oslo, Norway; Health Systems Research Unit, Medical Research Council of South Africa; 5 Norwegian Knowledge Centre for the Health Services, P.O. Box 7004, St. Olavs plass, N-0130 Oslo, Norway; Section for International Health, Institute of General Practice and Community Medicine, Faculty of Medicine, University of Oslo, Norway

## Abstract

This article is part of a series written for people responsible for making decisions about health policies and programmes and for those who support these decision makers.

Policymakers and those supporting them often find themselves in situations that spur them on to work out how best to define a problem. These situations may range from being asked an awkward or challenging question in the legislature, through to finding a problem highlighted on the front page of a newspaper. The motivations for policymakers wanting to clarify a problem are diverse. These may range from deciding whether to pay serious attention to a particular problem that others claim is important, through to wondering how to convince others to agree that a problem *is* important. Debates and struggles over how to define a problem are a critically important part of the policymaking process. The outcome of these debates and struggles will influence *whether* and, in part, *how* policymakers take action to address a problem. Efforts at problem clarification that are informed by an appreciation of concurrent developments are more likely to generate actions. These concurrent developments can relate to policy and programme options (e.g. the publication of a report demonstrating the effectiveness of a particular option) or to political events (e.g. the appointment of a new Minister of Health with a personal interest in a particular issue). In this article, we suggest questions that can be used to guide those involved in identifying a problem and characterising its features. These are: 1. What is the problem? 2. How did the problem come to attention and has this process influenced the prospect of it being addressed? 3. What indicators can be used, or collected, to establish the magnitude of the problem and to measure progress in addressing it? 4. What comparisons can be made to establish the magnitude of the problem and to measure progress in addressing it? 5. How can the problem be framed (or described) in a way that will motivate different groups?

## About STP

*This article is part of a series written for people responsible for making decisions about health policies and programmes and for those who support these decision makers. The series is intended to help such people ensure that their decisions are well-informed by the best available research evidence. The SUPPORT tools and the ways in which they can be used are described in more detail in the Introduction to this series *[1]. *A glossary for the entire series is attached to each article *(see Additional File 1). *Links to Spanish, Portuguese, French and Chinese translations of this series can be found on the SUPPORT website (http://www.support-collaboration.org). Feedback about how to improve the tools in this series is welcome and should be sent to: STP@nokc.no.*

## Scenarios

Scenario 1: You are a senior civil servant and have been asked to submit a briefing note to the Minister about a health system problem in which she has a personal interest, namely that many of her constituents and family members say that they can’t find a primary healthcare physician. You are concerned about whether the current draft of the briefing note prepared by a junior policy analyst does justice to the problem.

Scenario 2: You work in the Ministry of Health and are preparing a briefing note about a health system problem. All that you have been told is that the problem is about many citizens not having access to primary healthcare providers and services.

Scenario 3: You work in an independent unit that supports the Ministry of Health in its use of research evidence in policymaking and are preparing a policy brief for the Ministry of Health on barriers to accessing primary healthcare. You want guidance on how to clarify the problem in a systematic and comprehensive way.

## Background

**Figure 1:**
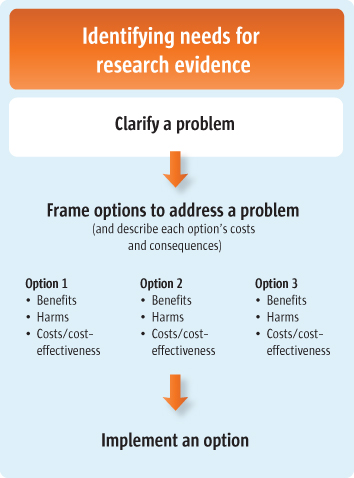
Clarifying evidence needs

For policymakers (Scenario 1), this article suggests a number of questions that they might ask their staff to consider when preparing a briefing note about a problem. For those who support policymakers (Scenarios 2 and 3), this article suggests a number of questions to guide the clarification of a problem based on the best available local and global evidence. This article is the first of three articles about clarifying evidence needs (see also Articles 5 and 6 [2,3]). Figure 1 outlines the processes involved in clarifying these needs.

Policymakers and those supporting them often find themselves in situations in which they need to decide how best to define a problem. They may have:

• Identified a problem through an explicit priority-setting process (the focus of Article 3) [4]

• Read about a problem in a report from a national statistical agency or from an independent researcher

• Been asked a tough question about a problem in the legislature or by someone living in their constituency

• Found a problem highlighted on the front page of a daily newspaper, or

• Identified a problem through their personal experience of a health system

Some of these situations lend themselves to the proactive assessment of a problem, or what some might call an issue or challenge. But most typically they place policymakers in a reactive mode.

The motivation for policymakers to clarify a problem may be informed by a consideration of:

• Whether to pay serious attention to a particular problem that others assert is important

• What factors contribute to a problem

• How to measure the magnitude of a problem (whether it is getting better or worse, and whether it is responding to particular policies or programmes)

• How to convince others to agree that a problem is important (or that a favoured way forward is the optimal one given *how* it addresses a particular problem), or

• How to address misperceptions or manage expectations among those who (erroneously, in the eyes of the policymakers) see the problem as important

Debates and struggles over how to define a problem are a critically important part of the policymaking process [5,6]. The outcome of these debates and struggles will influence *whether* (and, in part, *how*) policymakers take action to address a problem.

Problems may come to light through:

• A focusing event

• A change in an indicator, or

• Feedback from the operation of a current policy or programme [7]

Focusing events are very common in the health sector because poor decision making may lead to extreme and often high-profile events such as illness and death. An example of a focusing event would be extensive newspaper coverage over a number of consecutive days of the provision of counterfeit prescription drugs and the deaths resulting from their use. A change in an indicator, though less dramatic, can also bring a problem to attention, particularly if it is a large change or it receives significant attention in a report or media release. A national statistical agency, for instance, may release a report that shows that nurses’ pay varies widely across a country and that this is contributing to nursing shortages in certain provinces. Or a problem may come to light through feedback from the operation of a current policy or programme. Informal feedback from a programme manager in charge of a provincial waiting-time reduction initiative might, for example, highlight the fact that the programme is failing to meet its target for wait-time reductions due to resource limitations.

However, not all problems that are brought to attention are deemed worthy of government action. A problem can be defined as warranting government action by:

• Comparing current conditions with values related to a ‘more ideal’ state of affairs

• Comparing performance with other jurisdictions, and

• Framing a subject in a different way (e.g. describing a problem as an impediment to achieve a national priority) [7]

Politicians from different political parties will reflect different values and interpretations related to what constitutes a ‘more ideal’ state of affairs. A Minister of Health might regard the performance of their own country’s health system favourably relative to another in a neighbouring country. But he or she might not do so when it is compared less favourably to other but equally appropriate international examples. Similarly, a cabinet may decide to take action if a particular problem is defined in terms of a lack of patient choice among healthcare providers (given that this could potentially become a source of frustration for voters), but *not* if a problem is defined in terms of a lack of interest on the part of physicians in joining clinics that use collaborative practice models (this issue might be perceived by them as being too far removed from the concerns of voters).

Efforts to clarify problems are more likely to result in action if they:

• Reflect an awareness of concurrent developments related to policy and programme options (e.g. the publication of a report demonstrating the effectiveness of a particular option), and

• Are influenced by concurrent political events (such as the appointment of a new Minister of Health who may have a personal interest in a particular issue) [7]

If a problem is not defined in a way that ‘fits well’ with what are perceived to be viable options, or if it does not fit with broader political events, it is very unlikely to reach a decision agenda. An option can be deemed to be a viable solution if it is technically feasible, fits with dominant values and the public’s current mood, and is acceptable both in terms of budget workability and likely political support or opposition [7]. Relevant political events can include swings in the public mood, changes in levels of support or opposition from interest groups, and changes to the governing party or prevailing legislative coalition [7].

## Questions to consider

The following questions can guide how to identify a problem and characterise its features:

1. What is the problem?

2. How did the problem come to attention and has this process influenced the prospect of it being addressed?

3. What indicators can be used or collected to establish the magnitude of the problem and to measure progress in addressing it?

4. What comparisons can be made to establish the magnitude of the problem and to measure progress in addressing it?

5. How can a problem be framed (or described) in a way that will motivate different groups?

### 1. What is the problem?

A problem may relate to one or more of the following:

• A risk factor, disease or condition

• The programmes, services or drugs currently being used to address a risk factor, disease or condition

• The current health system arrangements within which programmes, services and drugs are provided, or

• The current degree of implementation of an agreed upon course of action (e.g. a policy or guideline)

The prevalence of a risk factor or the burden of a disease or condition in a province or country (e.g. incidence rate, prevalence rate, mortality rate) may constitute a problem. But more often, such issues are the manifestation of a problem: their *cause* is the real problem that needs to be addressed. The problem may instead lie with the programme or service, or relate specifically to the suitability of a drug that is currently being used to address a risk factor, disease or condition. Ineffective programmes, services or drugs may, for example, be in use to prevent or treat the risk factor, disease or condition.

Alternatively, a problem may be rooted in current health system arrangements within which programmes, services and drugs are provided. Potential problems may lie with governance arrangements/structures. These can include:

• Who has policy (e.g. regulatory), organisational, commercial and professional authority and accountability over particular programmes

• The services and drugs or the parts of the health system within which the programmes are located

• The services and drugs provided

• How authority is discharged, and

• How people who exercise authority are held accountable

Potential problems may also be rooted in financial arrangements. Such arrangements may affect who finances (i.e. who pays for) particular programmes, services and drugs and the parts of the health system within which these are provided, or how organisations are funded to deliver them. It may also relate to how professionals are remunerated to provide programmes, services or drugs, whether patients/consumers are offered incentives to use them, and how resources are allocated to them. Further, problems may be linked to current delivery arrangements. These may include: who is targeted by particular programmes, services and drugs, who they reach (or who accesses and uses them), who provides them and how, where they are they provided, what information and communication technology is used to provided them, and what safety and quality systems are provided. The taxonomy of governance, financial and delivery arrangements is addressed further in Article 7 in this series [8].

Finally, a problem may be rooted in the *degree* of implementation of an agreed course of action about a programme, service or drug, or else an agreed course of action about the health system arrangements within which these are provided. A problem, for example, may already have been defined and a policy introduced to address it, but the policy may not yet have been translated into action. In this instance, one approach to identifying the problem is to identify potential barriers to implementation at one or more of four levels:

1. The healthcare recipient and citizen level (e.g. citizens are unaware that they can access a programme, service or drug free of charge)

2. The healthcare provider level (e.g. health workers do not fully adhere to national policies and guidelines)

3. The organisational level (e.g. organisations do not manage the performance of their staff), and

4. The system level (e.g. policies are not enforced).

The identification of barriers to implementation is the focus of Article 6 in this series [3].

Policymakers and those who support them need to determine the causes of a problem. These problems may be related to: one or more of a risk factors; a disease or condition; the programmes, services or drugs currently being used; the current health system arrangements; or the current degree of implementation of an agreed upon course of action. Doing so can be an iterative process. What at first glance may appear to be a seemingly unrelated issue, such as disincentives to manage chronic disease proactively in primary healthcare, may actually be the very problem that needs attention. Table 1 illustrates how this simple framework can be used to clarify a problem, using malaria treatment in sub-Saharan Africa as an example.

**Table 1:** Clarifying the problem underpinning the lack of widespread use of the recommended malaria treatment

Members of the Evidence-Informed Policy Networks (EVIPNet) in ten sub-Saharan African countries identified the problem of the lack of widespread use of the recommended artemisinin-based combination therapy (ACT) to treat malaria in their respective countries. The following framework of four questions (and relevant sources of data and research evidence) [11] was used to clarify this problem:
• Does the problem relate to a risk factor, disease or condition?
◊ Incidence of (and death rates from) uncomplicated falciparum malaria, by age (including separately for infants), sex (including separately for pregnant women and lactating women), HIV status, malnutrition status, and socio-economic status
• Does the problem relate to a programme, service or drug currently being used to address a risk factor, disease or condition?
◊ Cure rates for, and drug resistance (or reduced drug sensitivity) to, ACT and other anti-malarial drugs, as well as the side effects and costs of the drugs
◊ The views and experiences of patients about particular anti-malarial drugs
• Does the problem relate to the current health system arrangements within which programmes, services and drugs are provided?
◊ Governance arrangements
– Regulations about which ACT and other anti-malarial drugs (i.e. drugs, dosage regimes, and packaging) can be registered/licensed for sale, how counterfeit or substandard drugs are safeguarded against, how patents for them and profits arising from them are handled, how they can be marketed, who can prescribe them and how, and who can sell or dispense them and how
– National treatment guidelines and/or the national malaria control policy about the first-line (and second-line) drug therapy recommended for uncomplicated falciparum malaria, as well as their dosage regimes/packaging, targeting for particular populations, and targeting for areas with particular characteristics
– National essential drugs list, particularly the list of anti-malarial drugs
◊ Financial arrangements
– Drug and dispensing fees for first-line drug therapy (and for ACT if this is not the first-line therapy) for uncomplicated falciparum malaria, including any subsidies for particular populations, remuneration arrangements for health works prescribing and dispensing ACT
– The views and experiences of patients about fees and subsidies and about financial incentives to promote adherence
◊ Delivery arrangements
– Access rates for first-line drug therapy (and for ACT if this is not first-line therapy) for uncomplicated falciparum malaria (i.e. who has access to someone who can dispense drug therapy)
– Coverage rates for first-line drug therapy (and for ACT if this is not first-line therapy) for uncomplicated falciparum malaria (i.e. who is dispensed which drug)
– Treatment patterns for uncomplicated falciparum malaria (i.e. who dispenses what, when, where and how, including whether treatment is part of the Integrated Management of Childhood Illness or other ‘horizontal’ programmes)
– Adherence patterns for the treatment of uncomplicated falciparum malaria (i.e. who takes what, when, where and how)
– Arrangements for surveillance, pharmacovigilance and the diagnosis and treatment of atypical cases
– The views and experiences of patients about particular providers (or delivery arrangements more generally)
• Does the problem relate to the current degree of implementation of an agreed-upon course of action?
◊ For example, regulations can only help to address a problem if they are acted upon throughout the health system. Regulations may exist about the registration/licensure, marketing, prescribing and dispending of ACT and other anti-malarial drugs. However, if the regulations are not enforced, there may be many counterfeit or substandard drugs in circulation, false statements may be made in drug advertisements, and untrained individuals may be prescribing or dispending ACT
The EVIPNet teams all concluded that the problem could be related to a risk factor, disease or condition, the programmes, services or drugs currently being used, the current health system arrangements and, in some cases, the current degree of implementation of an agreed-upon course of action. This had important implications for which options were considered appropriate to address this multi-faceted problem.

Policymakers and those supporting them could gain additional insights into this component of problem clarification from the fields of complexity theory, complex adaptive systems, and soft systems methodology. Examples of relevant resources are provided at the end of this article.

### 2. How did the problem come to attention and has this process influenced the prospect of it being addressed?

Identifying a problem is often only the beginning of the process. Typically, a great deal of work will still need to be done in order to clarify a problem in a way that confirms whether or not there is a need to address it. If there is a need, it will also be necessary to build the support required to address it. Understanding how a problem first came to attention can be an important initial step in the process of clarification. As outlined in the Background section in this article, problems typically come to light through:

• A focusing event

• A change in an indicator, or

• Feedback from the operation of current policies and programmes

Key policymakers may (or may not) agree whether a problem warrants attention at the early stages of the problem-clarification process. Table 2 illustrates how the question discussed here in this sub-section (together with three additional questions) can be used to clarify a problem once it has been related to one or more of: a risk factor, disease or condition; the programmes, services or drugs currently being used; the current health system arrangements; or the current degree of implementation of an agreed upon course of action.

**Table 2:** Clarifying the problem underpinning high rates of medication error

Questions 2-5 which were discussed earlier in this article can be used to clarify a problem once it has been related to one or more of the following: a risk factor, disease or condition, the programmes, services or drugs currently being used, the current health system arrangements and the current degree of implementation of an agreed upon course of action. Consider the following example of the problem of high rates of medication error:
• How did the problem come to attention and has this process influenced the prospect of it being addressed?
◊ The problem of medical error may come to attention through a focusing event (e.g. a child dies because a doctor prescribes the wrong drug dosage), a change in an indicator (e.g. there is a dramatic increase in the number of reported errors in a given month) or feedback from the operation of current policies and programmes (e.g. an evaluation report identifies more types of medication errors than have been routinely measured)
◊ An evaluation report may identify that one possible factor contributing to a problem is the lack of clear boundaries of the scope of practice between doctors, nurses and pharmacists, which makes accountability for prescribing, dispensing, administration and chart documentation unclear
◊ The same report may propose that the problem be turned into a statement of purpose that can be used to engage a diverse array of stakeholders. For example, policymakers may prefer to speak about how their country will become a leader in patient safety, rather than referring to current patient safety problems
• What indicators can be used or collected to establish the magnitude of the problem and to measure progress in addressing it?
◊ Policymakers may identify that no indicators are currently being measured accurately at the national level but that they are interested in starting to accurately measure both the number of medication error reports per quarter and the number of ‘near misses’ per quarter. Collecting such data would allow them to set a target level for the indicator
• What comparisons can be made in order to establish the magnitude of the problem and to measure progress in addressing it?
◊ Policymakers may identify that they would like to make four types of comparisons:
– Comparisons over time within the country
– Comparisons to other appropriate comparator countries
– Comparisons against a target to be set as part of a national patient safety strategy
– Comparisons against what a national consumer association has said it would like to see
◊ Ideally a search for administrative database studies or community surveys would allow the policymakers to identify at least some existing research evidence and allow them to make immediate comparisons
• How can a problem be framed (or described) in a way that will motivate different groups?
◊ Policymakers may find that:
– Pharmacists respond to the language used to describe a medication error
– Consumer groups respond to a stated purpose of achieving, for example, a 50% reduction in medication errors
– Regulators engage when the lack of clear boundaries between the scope of practice of healthcare providers is discussed as an important feature of the problem
– Hospital staff may respond positively when told of a plan to collect an indicator that identifies under-reporting in a way does not penalise units or departments who support full disclosure
– Hospital executives may engage most fully when comparisons are made among their facilities
◊ Ideally a search for qualitative studies would allow the policymakers to grasp the different meanings that different groups attach to a problem

If key policymakers *do* agree that a problem warrants attention and that they want to stake out a claim for what they would like to achieve in addressing the problem (e.g. through a statement of purpose or a goal), this will often leave little time to clarify the problem accurately. Before long, it may be necessary to move on to the specifics of considering how the options should be framed.

It is possible though that a focusing event could, on closer examination, turn out to be a significant aberration rather than reflecting a widespread problem. Similarly, an indicator may be found to have been poorly measured or not adjusted for seasonal variation. Or an internal report about the operation of current policies and programmes may, when read more closely, contain significant errors of interpretation. It may also be the case that policymakers erroneously link a problem to programmes, services or drugs currently being used when, in reality, the actual problem may lie elsewhere.

Alternatively, key policymakers may quickly decide that a problem does not warrant attention. They may focus on addressing misperceptions or managing expectations among those who first brought the problem to attention. In the interim, those supporting such policymakers may conduct a preliminary review and conclude that the problem *is* significant. In this case key policymakers will be left with the difficult task of having to make an argument for re-opening an issue that has been effectively closed – perhaps even in a highly visible way.

### 3. What indicators can be used or collected to establish the magnitude of the problem and to measure progress in addressing it?

Depending on how a problem first comes to attention, it may or may not be necessary to examine closely which indicators related to a problem are currently being measured (or can and should be measured) accurately. If, for example, a problem comes to attention through a change in an indicator that is already known to be highly reliable, giving further attention to other indicators may not be needed. On the other hand, if a problem comes to attention through a focusing event, further work would be necessary. In such cases:

• Community surveys and vital registries are examples of good sources of indicators about a risk factor, disease or condition

• Healthcare administrative data (or what are sometimes called health management information systems), monitoring and evaluation data, community surveys, and healthcare provider surveys can be good sources of indicators about the programmes, services and drugs currently being used

• Legislation, regulation, policies, drug formularies, and policymaker surveys can be good sources of indicators about governance arrangements

• Health expenditure surveys and healthcare provider surveys can be good sources of indicators about financial arrangements

• Healthcare administrative data can be good sources of indicators about delivery arrangements, and

• Community surveys and healthcare provider surveys, as well as healthcare administrative data, can be good sources of indicators about the current degree of implementation of an agreed upon course of action

Disaggregated data, such as data by ethnicity/culture, gender or socio-economic status, can often be particularly helpful in clarifying whether a problem is widespread or particularly pronounced in some groups. Article 11 in this series addresses how to find and use local evidence, and Article 10 describes a categorisation scheme for groups which could be considered when incorporating equity-based approaches within the process of problem clarification [9,10].

### 4. What comparisons can be made to establish the magnitude of the problem and to measure progress in addressing it?

While indicators can provide policymakers with some sense of the magnitude of a particular problem, implicit or explicit comparisons are what truly establish whether a problem is big or small, whether it is getting better or worse, or whether it appears amenable to change. At least four key types of comparisons can be made:

• *Comparisons over time within a country*: can help to establish whether a problem is getting better or worse. If corrective actions have already been taken, such comparisons can help to determine whether a problem appears amenable to change

• *Comparisons between countries and other appropriate comparators* (where the data are comparable): can help to establish whether a problem is big or small and what targets could be achievable, and help to mobilise support for addressing a problem

• *Comparisons against plans*: (e.g. national targets and the Millennium Development Goals) can help to mobilise support for addressing a problem, and

• *Comparisons against what policymakers and/or stakeholders predicted or wanted*: can also help to mobilise support for reaching goals

While clarifying a problem relies extensively on local data, research evidence can often provide comparisons that have been conducted in a systematic and transparent way. Healthcare administrative database studies and community surveys, for example, which are often published in research literature, can help to clarify a problem and appropriate targets and mobilise support. Such studies can be highly useful to policymakers in addressing misperceptions or managing expectations. They can also be used to develop or refine a statement of purpose. For example, policymakers may want to change the trajectory of an existing indicator or measure a new indicator in ways that permit comparisons. Article 11 in this series provides approaches to finding and using local evidence [9]. Table 3 also provides tips for finding healthcare administrative database studies and community surveys.

**Table 3:** Finding research evidence about a problem

While much of the task of problem clarification involves finding and using local evidence (the subject of Article 11 in this series), published administrative database studies and community surveys can provide insights about comparisons [9]. Qualitative studies can also provide insight into alternative framings for a problem.
The first set of steps involved in finding such studies includes:
• Drawing up a list of words or phrases that capture the problem (e.g. medication error, scope of practice), synonyms for each problem and factor (e.g. drug near-misses, professional regulation), and alternative spellings for each synonym (e.g. medication, medications)
• Deciding whether systematic reviews (the subject of Article 7) or single research studies are the focus of the search [8], and
• Providing any additional details that limit the search (e.g. children, adults)
The second set of steps includes:
• Choosing those words and phrases that would *all* need to be present in order for the article to be identified (e.g. medication error, systematic review, and children), connecting them with ‘and’, and putting them in brackets, and
• Choosing those words and phrases for which only one would need to be present (e.g. medical error and its synonyms), connecting them with ‘or’, and putting them in brackets, and
• Connecting both sets of brackets using ‘and’
The third set of steps includes:
• Using the Internet to access the health-related database, PubMed. This database contains a ‘hedge’ (i.e. a validated search strategy or filter) for the types of studies of interest here [12]
• Clicking on ‘special queries’ in the left task bar
• Clicking on ‘health services research’ queries
• Entering the words and phrases, as well as the Boolean operators (‘and’/‘or’) in the search field, and
• Clicking ‘process assessment’ or ‘outcomes assessment’ for administrative database studies and ‘qualitative research’ for qualitative studies
This approach increases the chances that the returned citations will be of the appropriate study type, though many other types of studies may be retrieved as well.

### 5. How can a problem be framed (or described) in a way that will motivate different groups?

How a given problem is categorised can have important consequences for the way groups may respond. Framing a problem in new or alternative ways is likely to result in the issues resonating in different ways among different groups. Canada, for example, has framed the field of study related to the social determinants of health most neutrally by referring to it as ‘population health’. In contrast, in the United States, the same field is often referred to as ‘disparities in health’ – a term that conveys the existence of differences but not necessarily unfairness. In the United Kingdom, the term ‘inequalities in health’ is commonly used. This term seems explicitly to convey unfairness, and it only gained political traction when a new governing party was elected in the 1990s with a goal of reducing unfairness within health and other sectors. Some groups may respond more actively to a negatively framed problem statement (e.g. “Our country has the highest infant mortality rate in the region”) while others may respond better to a positively framed statement of purpose (e.g. “Our country will, within five years, achieve the national health goals related to infant mortality”).

Some groups may rally around issues related to a particular disease or condition (e.g. rapidly rising rates of cardiovascular disease). Others may rally around one or more risk factors (e.g. smoking, diet, exercise or housing and working conditions). Even groups with a similar focus may be attracted to different indicators related to the same problem. Some may be motivated more by ‘hard’ indicators such as mortality. But others may be motivated by ‘soft’ indicators such as self-reported health status. Particular groups may be motivated only by indicators from the health sector, such as health-related quality of life. Other groups, in contrast, may be motivated by indicators from non-health sectors that can have an influence on health and healthcare, such as employment status. The importance of comparisons can also vary by group, with some groups more interested in a narrowly defined group of peers that share a range of key characteristics (such as large university-affiliated teaching hospitals), and others more interested in the full spectrum of organisations providing similar types of healthcare (such as all hospitals).

Qualitative research can shed light on the meanings that individuals or groups attach to a particular problem, the indicators used to measure it, and the comparisons made to establish its importance. Table 3 provides tips for finding this type of research. Conversations with different groups and available qualitative research can help policymakers identify which framings of a problem (or purpose) can best mobilise support among different groups to address a problem. A key challenge, however, is ensuring that the alternative framings being considered are consistent with the problem, as determined through the type of systematic analysis described above.

## Conclusion

Problem clarification can all too easily be skipped over entirely, or else done too rapidly, or in too cursory a way. It may also not be done iteratively when additional data and research evidence are found regarding indicators and comparisons, or when policies and programmes encounter challenges or fail to yield results. Any such failures in problem clarification may mean that further resource investments based on existing conceptions of a problem will be misguided. Close attention should be paid therefore to indicators, comparisons and alternative framings to ensure that decisions about which particular problem to focus on are well-informed. The process of clarifying a problem will influence decisions about which particular options warrant serious consideration based on *how* they address a problem.

## Resources

### Useful documents and further reading

- Kingdon JW: *Agendas, Alternatives, and Public Policies,* 2 edn. New York, USA: Longman; 2003, pp. 90-115.

- Rosenhead J, Mingers J (Eds): *Rational Analysis for a Problematic World Revisited:. Problem Structuring Methods for Complexity, Uncertainty and Conflict.* Chichester, UK: John Wiley & Sons Ltd.; 2001; pp 61-2.

- Stone D: *Policy Paradox:* The Art of Political Decision Making. New York: W. W. Norton and Company; 1997.

- Sweeney K. Griffiths F (Eds). *Complexity and Healthcare.* An Introduction. Oxford, UK: Radcliffe Medical Press; 2002, pp. 100.

### Links to websites

- Program in Policy Decision-Making (PPD)/Canadian Cochrane Network and Centre (CCNC) database: http://www.researchtopolicy.ca/search/reviews.aspx – Source of a taxonomy of governance, financial and delivery arrangements within health systems where problems may be located, as well as systematic reviews of administrative database studies, community surveys, and qualitative research addressing health system arrangements

PubMed Health Services Research (HSR) Queries: 

http://www.nlm.nih.gov/nichsr/hedges/search.html – Source of ‘hedges’(i.e. validated search strategies) to identify administrative database studies and community surveys that can help to put a problem in comparative perspective and to identify qualitative studies that can help to frame problem in ways that resonate with different stakeholders

## Competing interests

The authors declare that they have no competing interests.

## Authors’ contributions

JNL prepared the first draft of this article. MGW, ADO, SL and AF contributed to drafting and revising it.

## Supplementary Material

Additional file 1 Glossary Click here for file
